# The Influence of Indirect Bonding Technique on Adhesion of Orthodontic Brackets and Post-Debonding Enamel Integrity—An In Vitro Study

**DOI:** 10.3390/ma16227202

**Published:** 2023-11-17

**Authors:** Agnieszka Nawrocka, Ireneusz Piwonski, Joanna Nowak, Salvatore Sauro, María Angeles García-Esparza, Louis Hardan, Monika Lukomska-Szymanska

**Affiliations:** 1 Department of General Dentistry, Medical University of Lodz, 251 Pomorska St., 92-213 Lodz, Poland; agnieszka.nawrocka@stud.umed.lodz.pl; 2 Department of Materials Technology and Chemistry, Faculty of Chemistry, University of Lodz, 163 Pomorska St., 90-236 Lodz, Poland; ireneusz.piwonski@chemia.uni.lodz.pl; 3 University Laboratory of Materials Research, Medical University of Lodz, 251 Pomorska St., 92-213 Lodz, Poland; joanna.nowak.1@umed.lodz.pl; 4 Dental Biomaterials, Preventive and Minimally Invasive Dentistry Departamento de Odontología, Facultad de Ciencias de la Salud, Universidad CEU-Cardenal Herrera C/Del Pozo ss/n, Alfara del Patriarca, 46115 Valencia, Spain; 5 Department of Therapeutic Dentistry, I.M. Sechenov First Moscow State Medical University, 119146 Moscow, Russia; 6 Department of Pharmacy, School of Health Sciences, Universidad Cardenal Herrera-CEU, CEU Universities, Elche, 03204 Alicante, Spain; maria.garcia2@uchceu.es; 7 Department of Restorative Dentistry, School of Dentistry, Saint-Joseph University, Beirut 1107 2180, Lebanon; louis.hardan@usj.edu.lb

**Keywords:** adhesion, APC brackets, ARI, ceramic brackets, etch-and-rinse technique, indirect bonding, SBS, shear bond strength

## Abstract

The increasing demand for orthodontic treatments due to the high prevalence of malocclusion has inspired clinicians and material scientists to investigate innovative, more effective, and precise bonding methods with reduced chairside time. This study aimed at comparing the shear bond strength (SBS) of metal and ceramic brackets bonded to enamel using the indirect bonding technique (IDB). Victory Series metal brackets (Metal-OPC, Metal-APC) and Clarity™ Advanced ceramic brackets (Ceramic-OPC) (3M Unitek, Monrovia, CA, USA) were bonded indirectly to extracted human premolars through the etch-and-rinse technique. A qualitative assessment of the enamel surface using microscopic methods was performed, and the amount of residual adhesive was reported as per the adhesive remnant index (ARI). Moreover, the bracket surface was evaluated with SEM-EDS. The highest SBS mean values were observed in the Ceramic-OPC group (16.33 ± 2.01 MPa), while the lowest ones were obtained with the Metal-OPC group (11.51 ± 1.40 MPa). The differences between the Metal-AOPC vs. Metal-APC groups (*p* = 0.0002) and the Metal-OPC vs. Ceramic-OPC groups (*p* = 0.0000) were statistically significant. Although the Ceramic-OPC brackets bonded indirectly to the enamel surface achieved the highest SBS, the enamel damage was significantly higher compared to that of the other groups. Thus, considering the relatively high bond SBS and favourable debonding pattern, Metal-APC brackets bonded indirectly may represent the best choice.

## 1. Introduction

Modern orthodontic practice should constantly pursue enhanced treatment standards with reduced chairside time. One of the most time-consuming procedures in orthodontics is the bonding of orthodontic brackets. It is well known that the ideal positioning of attachments requires a skilled operator with a high level of precision. Thus, the indirect bonding technique (IDB) may satisfy such a need for reduced chairside time and the proper placement of brackets on dental enamel. In this method, brackets are firstly positioned on the model outside the oral cavity and then transferred, using a custom-made splint, intraorally at a specific position [1,2,3]. A further simplification method is based on the pre-coating of the brackets with adhesive (APC), which, in contrast to conventional operator-coated brackets (OPC), requires a shorter application time because the base of the bracket is covered with an optimal amount of orthodontic adhesive [4,5].

The first APC orthodontic brackets were introduced in 1991 (Mini Unitwin, 3M, Unitek) [4,6,7]. At that time, such ready-to-use brackets with the bases covered with a composite were chemically cured on the enamel using orthodontic adhesives (two-paste systems), which was a huge step forward in improving the bonding procedure [4,5]. In the 1990s, the working time was limited by the chemically cured material, leading to imperfections in the bracket position and more residual adhesive around the bracket base. Mini Unitwin APC brackets overcame such limitations [1,2,3]. Indeed, the next generations of APC brackets were APCP (APC Plus) (which were tolerant to moisture and released fluoride) and APCF (a flash-free adhesive precoated system) in 2014, along with a low-viscosity resin that prevented material excess around the bracket base [8,9,10]. Indeed, once the attachment was positioned properly, the operator only needed to press on the bracket and the material could form a transparent sealing layer between the base and enamel, reducing microleakage [11,12].

APC brackets are distributed in two variants: metal and ceramic, similarly to their OPC counterparts. Metal brackets are made of austenitic stainless steel and have a foil mesh base to improve their retention. On the contrary, ceramic brackets, also described as “aesthetic”, are composed of aluminium oxide (alumina). Depending on their internal structure, two types of ceramic brackets can be distinguished—polycrystalline (with multiple particles of Al_2_O_3_) and monocrystalline (made of a single crystal). Despite having such a similar chemical composition, they possess differences in terms of the manufacturing process and their internal structure, which determine their physical properties. Polycrystalline brackets are fabricated using ceramic injection moulding technology (CIM)—multiple crystals are mixed with an agglutinative agent and melted above 1800 °C [13,14]. Over this temperature, the binder is burnt and the liquid mixture containing Al_2_O_3_ is injected into the mould. During the sinterization and cooling down processes, the final bracket shape is obtained [13]. Monocrystalline brackets (sapphire) are produced by means of the slow and controlled crystallization of Al_2_O_3_ (at a temperature of 2100 °C) to obtain a single crystal rod. The product is milled to achieve the desired bracket shape. Polycrystalline brackets are not transparent but are more resistant to fracturing in comparison to sapphire ones. This is due to the fact that in the former, the boundaries between crystal grains serve as a delimiter for the propagation of cracks [14]. The etching and bonding protocol of both types of ceramic brackets is similar to that of OPC brackets. The enamel surface is prepared through etch-and-rinse (ER) or self-etch (SE) bonding protocols. According to the literature, the total bonding time using the SE technique is shorter, but SBS values are often lower in comparison to those when using the ER technique [5,15,16,17,18].

A combination of APC brackets with IDB seems to be an effective and efficient bonding method. However, the reduction in chairside time should not come at the expense of the quality of the adhesion; therefore, the performance of this solution should be investigated [5]. 

This study aimed at comparing the shear bond strength (SBS) of Metal-OPC, Metal-APC, and Ceramic-OPC brackets bonded indirectly to an enamel surface. The qualitative assessment of the enamel surface using microscopic methods was performed, and the amount of residual adhesive was reported as per the adhesive remnant index (ARI). The null hypothesis was that the combination of Metal-APC brackets with IDB would result in a significantly lower SBS in comparison to that of metal brackets coated by an operator. 

## 2. Materials and Methods

### 2.1. Sample Preparation

Thirty-two human premolars extracted for orthodontic purposes were used in this study [19]. Institutional Ethical Committee approval was obtained for this study (RNN/147/19/KE).

All specimens were analysed under BX51optical microscope (Olympus, Tokyo, Japan) (20×) to check the enamel’s condition before etching and bonding procedures. No enamel damage, scratching, or carious lesions were detected in the teeth used in this study. The specimens were mounted on cylindric blocks made of self-cured acrylic resin (Duracryl Plus, SpofaDental, Jičín, Czech Republic). The specimens were cleaned with a water-cooled rotating brush mounted on a contra-angled handpiece. Non-fluoride paste (Clean Polish, Kerr, Grand Prairie, TX, USA) was chosen to avoid possible interaction of fluoride with enamel surface and undermining the etching process. Then specimens were rinsed and dried thoroughly with dental air spray syringe. The lingual surfaces of the specimens were positioned on the acrylic blocks so that the buccal surfaces remained accessible for the bonding procedures.

### 2.2. Preparation of Transfer Trays

Polivinylsiloxane impressions (Express STD VPS Impression Material, 3M ESPE, St. Paul, MN, USA) were taken from each tooth. These were subsequently poured using type IV gypsum (Kromotypo 4, LASCOD, Sesto Fiorentino, Italy). All casts were covered with separating/insulation liquid (Isocera, Bego, Bremen, Germany) and left to dry for 24 h. Brackets were positioned on the casts and light-cured for 3 h (Curing Pen; Eighteeth, Changzhou, China), using a 5 W high-power blue LED (with a 380–515 nm wavelength). Transfer trays were fabricated using the Lumaloc + Emiluma system (Ultradent Products Inc., South Jordan, UT, USA)—polivinylsiloxane and transparent silicone were used for indirect bonding [1,20].

**Figure 1:**
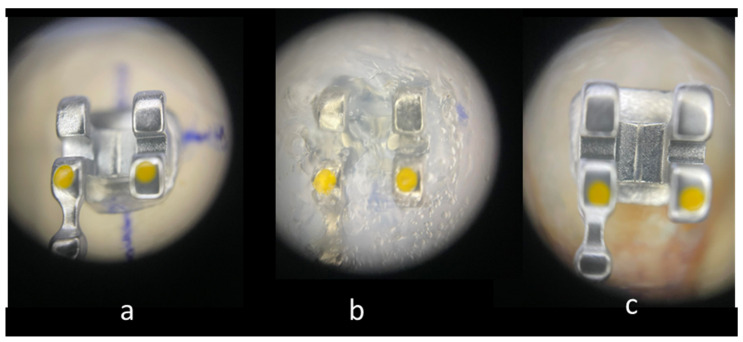
Main stages of IDB: (20×). (**a**) Bracket positioned on the cast. (**b**) Bracket immersed in the transparent polyvinylsiloxane transfer tray. (**c**) Bracket bonded to enamel surface.

The brackets on the casts were covered with Emiluma, followed by immediate application of Lumaloc according to the manufacturer’s instructions. Then the casts were placed in water at room temperature for 30 min. And finally, the transparent transfer trays were removed from the casts with the brackets inside (Figure 1).

### 2.3. Adhesive Preparation and Bracket Placement

Thirty specimens were randomly selected and divided into three study groups (n = 10): operator-coated metal brackets (Metal-OPC), adhesive precoated metal brackets (Metal-APC), and operator-coated ceramic brackets (Ceramic-OPC). In OPC groups, the bracket bases were manually covered with orthodontic adhesive. The amount of adhesive was standardized—2 mm of material measured with endodontic ruler was taken out using a syringe with a dental paddle and spread on the bracket base to cover the whole surface (the amount of 2 mm was established as sufficient to cover all of the bracket base with a thin, uniform layer). In Metal-APC group, brackets were simply taken out from the blister—the optimal amount of orthodontic adhesive was provided by the manufacturer. Brackets were positioned and pressed firmly into place with the uniform force—300 g measured with Dontrix dynamometer (Acmedent, Concord, ON, Canada) [21,22]. All brackets were indirectly bonded to the enamel surface using the protocol described in Table 1. 

The bonding procedures were performed using the materials presented in Table 2. Sondhi Rapid Set System (3M, Unitek, USA) was used as a gold standard of orthodontic resin indicated for indirect bonding procedure [23,24,25]. Sondhi resin is characterized by increased viscosity for better performance in the indirect technique. An additional component, 5% silica filler, increases its ability to fill the rough, retentive bracket base or enamel surface [1]. After application of Sondhi two-component resin (resin A on the bracket base; resin B on the enamel surface), the transfer tray was placed on the tooth and left for 2 min, until the end of polymerization process. The characteristics of brackets are depicted in Table 3. To obtain the complementarity of our materials and coherence of our methodology, brackets and adhesives produced by one manufacturer were applied. Ceramic APC brackets were not included in this study because of the differences in adhesive resin composition (Clarity Advanced brackets were available only in APC Flash-Free version and could not be compared with Metal-APC-Plus brackets in terms of post-debonding adhesive remnants).

### 2.4. SEM of the Bracket–Enamel Interface 

Two additional specimens (one metal and one ceramic bracket) that were not included in the SBS test were used to perform the SEM ultra-morphology analysis before debonding in order to illustrate the bracket–enamel interface (APC bracket was excluded from this observation because its bracket base structure is identical to that of Metal-OPC bracket). The specimens were dried and coated with 10 nm layer of gold using Q300TT sputter coater (Quorum Technologies, Wellington, ON, Canada) to provide optimal conditions for microscopic observation without sample charging [26].

### 2.5. Shear Bond Strength 

Specimens of study groups were stored in distilled water for 24 h at 37 °C. Then, thermocycling (5000 thermocycles, water baths of 5 °C and 55 °C, and a dwell time of 60 s) was performed to simulate intraoral aging of materials. The Zwick/Roell Z020 universal testing machine (Zwick-Roell, Ulm, Germany) was used for bracket debonding (1 mm/min crosshead speed). The load was parallel to the bracket base and the shear force causing the fracture at the enamel–bracket interface was registered (in Newtons). Shear bond strength (SBS) expressed in megapascals (MPa) was calculated as follows: SBS [MPa] = shear force [N]/bracket base area [mm^2^]. 

### 2.6. ARI Score 

After debonding, specimens were analysed via optical microscope (20×) using Årtun’s ARI scoring system [24]. Bracket bases were also evaluated under optical microscope with bracket adhesive remnant index (BARI) [25] (Table 4).

### 2.7. SEM-EDS of Specimens after SBS

Tooth specimens with ARI = 3 were further analysed via scanning electron microscope (SEM, NovaNanoSem 450, FEI, Hillsboro, OR, USA) to demonstrate the most enamel-safe pattern of adhesive failure at different magnifications (65–2000×). Unambiguous impression of the whole bracket base on enamel surface was a simple selection criterion. These specimens were prepared as described above. 

Moreover, randomly chosen brackets with BARI = 5 were subject to a further SEM-EDS analysis (EDAX/AMETEK, Materials Analysis Division, Model Octane Super, Mahwah, NJ, USA). Such type of adhesive failure (when material covers whole bracket base) is associated with the highest risk of enamel damage during debonding. Thus, the specimens with BARI = 5, apart from typical bracket and resin components, may also contain enamel-borne elements. As an indicator of possible enamel damage, the incidence of calcium in the chemical composition of brackets was assessed. 

### 2.8. Statistical Analysis

Results were analysed using a one-way analysis of variation (ANOVA) to determine the statistical significance of SBS values. Statistical significance was defined as *p* < 0.05 and calculated minimal sample size was up to 10 (test power = 0.98). Tukey’s post hoc test was used to determine which particular differences between groups of means were significant. To confirm the statistical significance of the collected ARI score, a non-parametric Kruskal–Wallis test was used. To assess the differences between the mean values of ARI in particular groups, post-hoc analysis with the Mann–Whitney U test was performed. Pearson correlation coefficient (PCC) was used confirm the correlation between SBS values and ARI scores in particular groups.

## 3. Results

### 3.1. SEM of the Bracket–Enamel Interface 

**Figure 2:**
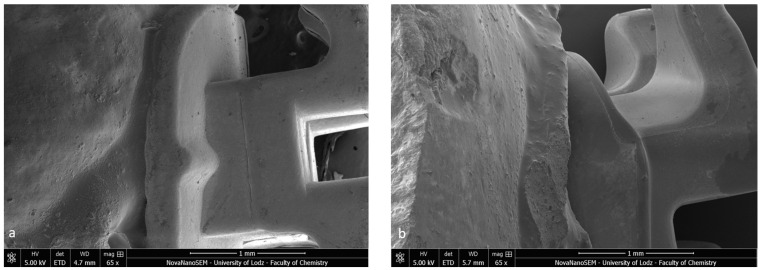
Bracket–enamel connection in SEM (65×): (**a**) Metal-OPC, (**b**) Ceramic-OPC.

**Figure 3:**
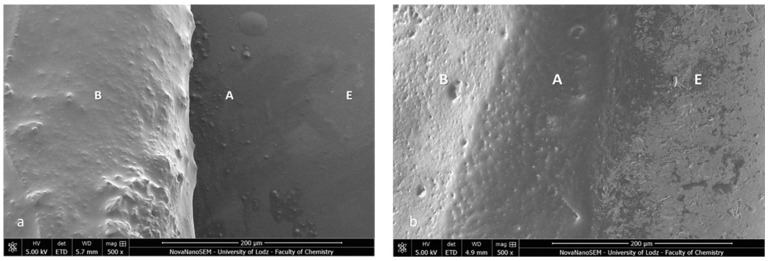
SEM image of the bracket–enamel interface in ER protocol (500×): (**a**) Metal-OPC (**b**) Ceramic-OPC. B—bracket base; A—orthodontic adhesive; E—etched enamel surface covered with adhesive resin.

Firstly, the SEM images of both specimens present a homogenous bonding interface at the bracket–enamel connection (Figure 2). The image presents a general overview of the Metal-OPC (Figure 2a) and Ceramic-OPC brackets (Figure 2b) with a typical structure, bonded to the etched enamel. Both types of brackets are manufactured in injection moulding technology; thus, the edge between the bracket base and the wings is not present. The cross-section exhibited no gaps or pores in the adhesive layer. The material appeared firmly connected with the retention area of the bracket base. At a high (500×) magnification, the adhesive layer between the two phases (the bracket and enamel) was clearly detected (Figure 3). 

**Figure 4:**
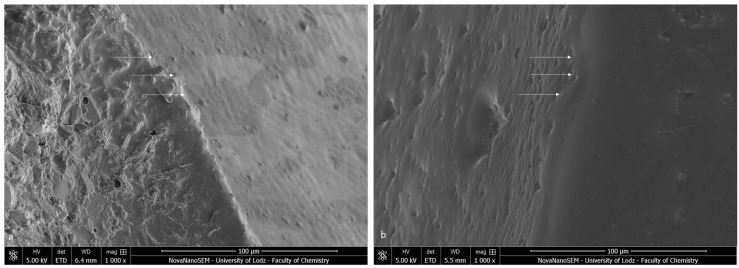
SEM image of the bracket–enamel interface in ER protocol (1000×): (**a**) Metal-OPC (**b**) Ceramic-OPC. Arrows represent the connection area.

The bracket bases created a firm connection with the orthodontic material. The light-cured adhesive covered and incorporated the base of the austenitic stainless-steel bracket (Figure 3a). The typical retentive mesh structure was not visible. In ceramic polycrystalline brackets (Figure 3b), a mechanical retention was created by a layer of microcrystals. The base uniformly was covered by the resin adhesive, and only crystalline grains are scattered in the material phase. The enamel did not present a characteristic post-etching pattern (with a selective dissolution of enamel prisms), because it was completely covered with adhesive resin. Using a magnification of 1000×, we confirmed the bonding integrity (Figure 4).

### 3.2. Shear Bond Strength 

**Figure 5:**
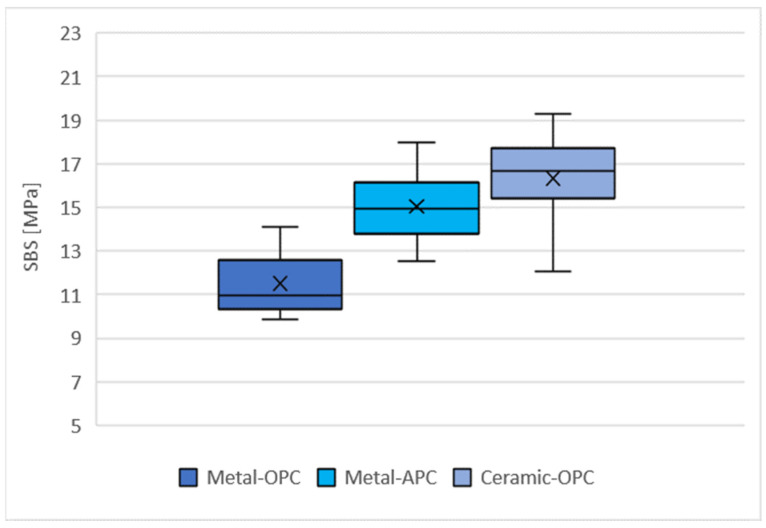
Mean SBS in tested groups (n = 10). SD: Metal-OPC = 1.4; Metal-APC = 1.64; Ceramic-OPC = 2.01, ×—mean value.

The distribution of the SBS values for the tested groups is presented in Figure 5. The highest mean SBS was obtained with the specimens in the Ceramic-OPC group (16.33 ± 2.01 MPa), while the lowest values were achieved in the Metal-OPC group (11.51 ± 1.40 MPa). The mean values of the SBS for the Metal-OPC group were significantly lower than those of the Metal-APC (*p* = 0.0002) and Ceramic-OPC (*p* = 0.0000) groups (Table A1, Appendix A).

### 3.3. ARI Score

**Figure 6:**
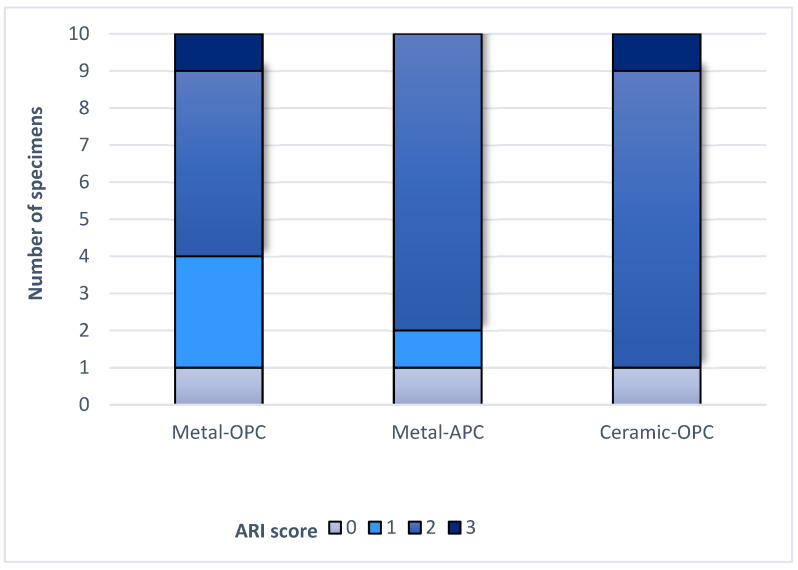
ARI score in tested groups.

**Figure 7:**
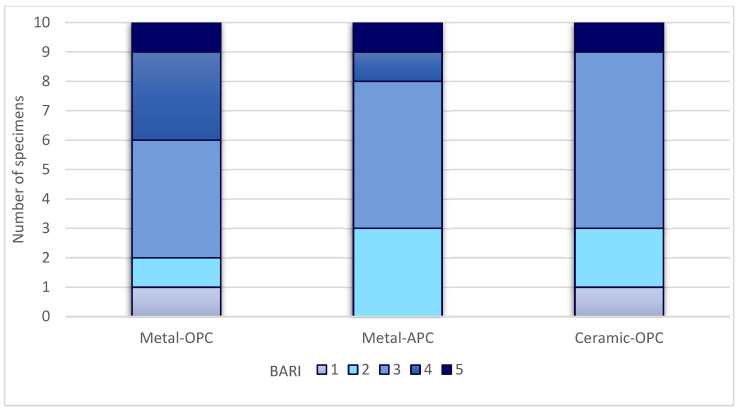
BARI score in tested groups.

The ARI values of the groups are presented in Figure 6 and the BARI values in Figure 7. 

The Kruskal–Wallis H test indicated that there was no significant difference in the ARI score between the groups (χ^2^ = 2.15, *p* = 0.341). According to the Mann–Whitney U test, the difference between the ARI values of the Metal-OPC and Metal-APC groups, the Metal-OPC and Ceramic-OPC groups, and the Metal-APC and Ceramic-OPC groups presented no statistical significance. In the Metal-OPC group, there was a significant negative relationship between the SBS and ARI (r = −0.95, *p* ≤ 0.01). In the Metal-APC group, there was also a very small negative correlation between the SBS and ARI (r = −0.05, *p* = 0.89), but it was not statistically significant. There was a significant very small negative relationship between the SBS and ARI in the Ceramic-OPC group (r = −0.764, *p* = 0.010).

### 3.4. SEM EDS Specimens after SBS

**Figure 8:**
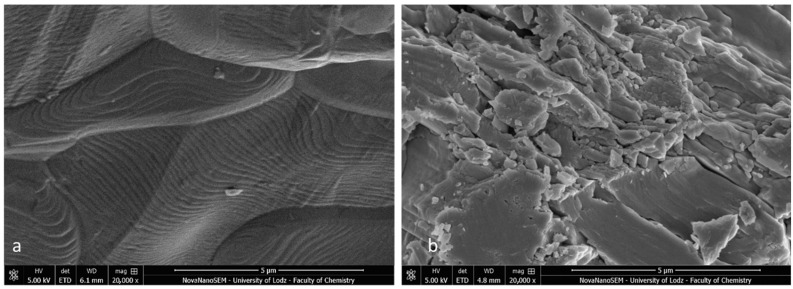
SEM image of bracket before bonding (not covered with adhesive); (**a**) Metal bracket; (**b**) Ceramic bracket (20,000×). Not applicable for APC brackets.

**Figure 9:**
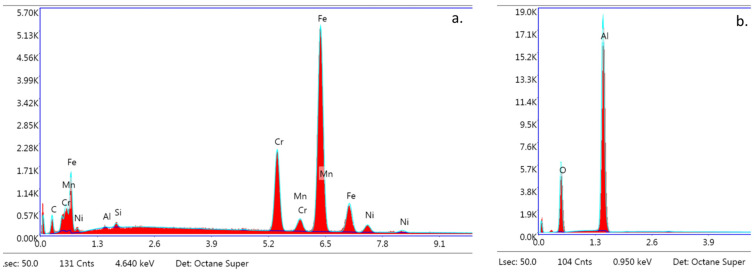
EDS analysis. Spectra obtained from s bracket before bonding (not covered with adhesive); (**a**) Metal bracket; (**b**) Ceramic bracket (20,000×). Not applicable for APC brackets.

The SEM images of the metal and ceramic brackets before bonding (not covered with adhesive) are presented in Figure 8a,b. The metal bracket (Figure 8a) made of an austenitic stainless-steel alloy presented a rough structure, which is quite characteristic in metal injection moulding manufacturing. The ceramic bracket (polycrystalline) image represents the structure of scattered crystals of Al_2_O_3_ in various shapes and sizes (Figure 8b). The EDS analysis confirmed the typical chemical composition of these brackets (Figure 9a,b; Table 5 and Table 6). The metal bracket consists of Fe (the dominant element); Cr, Ni, C, and Mn, which are typical stainless still alloy components; and minimal amounts of Si, Al, and P (Figure 9a). The polycrystalline bracket showed only two dominant components: Al and O (Figure 9b).

**Figure 10:**
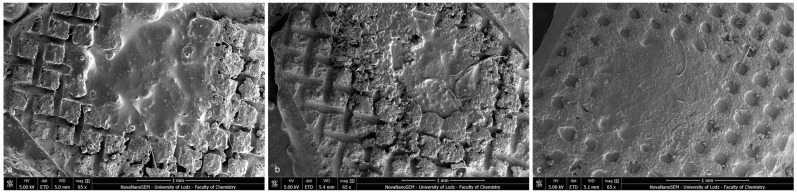
SEM images of tooth specimens with ARI scores of 3 (65×): (**a**) Metal-OPC, (**b**) Metal-APC, (**c**) Ceramic-OPC.

During our SEM observation, the specimens with ARI scores of 3 had enamel surfaces that were completely covered with adhesive residues and a clear “foot-print” of the bracket base (Figure 10a–c). The characteristic rectangular prominences correspond to the openings of the foil mesh metal bracket base. Linear, criss-crossing hollows represent the mesh wire gauge (Figure 10a,b). The regular apertures presented in Figure 10c are the stamp of mechanical and micromechanical crystalline elements in the ceramic bracket base. The central part of each bracket impression has a blurred image of the adhesive material (associated with the area of pressure during the bracket positioning and bonding).

**Figure 11:**
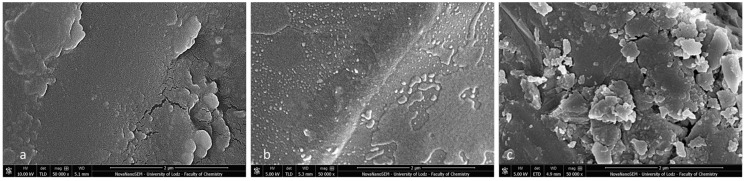
SEM observation of brackets with BARI scores of 5. (**a**) Metal-OPC, (**b**) Metal-APC, (**c**) Ceramic-OPC. Mag. 50,000×.

**Figure 12:**
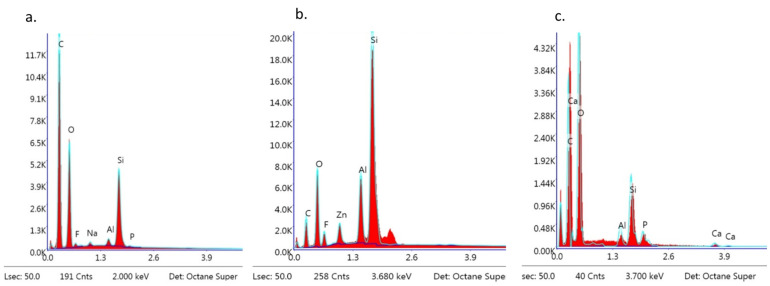
EDS observation of brackets with BARI scores of 5. (**a**) Metal-OPC, (**b**) Metal-APC, (**c**) Ceramic-OPC.

Post-debonding brackets with BARI scores of 5 were also qualified for SEM (Figure 11a–c) and EDS analyses (Figure 12a–c). The metal-OPC bracket base obtained a smooth surface without visible irregularities or retentive apertures (Figure 10a). The whole area was covered with a homogeneous layer of adhesive. The image of the metal-APC mesh base was not revealed, because of the presence of orthodontic material (Figure 10b). The surface was more irregular, with the presence of scattered elements in the material’s filler. The ceramic-APC bracket base showed diffused and well-disturbed crystalline elements embedded in adhesive resin (Figure 11c).

The EDS analysis revealed the following dominant elements in all of the samples: Si, C, O, and trace elements such as Na, Al, and P. Ca and F were also detected (Figure 12a–c).

## 4. Discussion

The effective bonding of brackets is a matter of time and cost optimization in orthodontic practice. The main reason for introducing APC brackets and the indirect bonding technique is to accelerate the workflow, eliminating operator errors. Since APC brackets were the first to be commercialised, numerous research reports were presented to demonstrate their potential predominance over conventional operator-coated brackets (OPC). Such research was mostly focused on the SBS, ARI scores, and the removal of adhesive remnants [11,12,27,28,29,30,31].

Hence, available scientific reports referring to SBS values are still inconclusive [8,27,28,29,30,31] and further investigations are required. In most studies, there was no statistically significant difference in SBS between APC and OPC brackets (Table 7) [8,27,28,29,31]. Only Ansari et al. [30] found a significantly higher SBS in OPC ceramic brackets in comparison to that of APC (Flash Free APC) brackets; the authors proposed differences in the stress distribution on the bracket base as a possible cause of such results.

In the present study, the Metal-APC brackets that were bonded indirectly showed significantly higher SBS values in comparison to those of the same type of brackets coated by the operator (Metal-OPC); thus, the null hypothesis must be rejected. The highest SBS values were observed in the specimens of the Ceramic-OPC group. A statistically significant difference was detected between the Metal-OPC and Ceramic-OPC brackets. According to the literature, APC brackets are the main choice in conventional orthodontic clinics, especially when implemented with the IDB, which offers strong bonding to enamel and improved bracket placement precision [11,12,27,28,29].

The reason for the higher SBS of ceramic brackets can be found in the chemical basis of the adhesion phenomenon. Orthodontic adhesive belongs to a group of polymers and can change the chain conformation as a response to interfacial interaction. Thus, the resin interacts differently with ceramic and metal surfaces. When the adhesive is in contact with a polycrystalline bracket surface, strongly absorbed and loosely absorbed polymeric chains are formed on the interface. Loosely absorbed chains are partially connected with the solid porcelain surface and increase the bonding strength; such an interaction does not occur with the metal bracket surface [32,33].

Referring to clinical differences, there is no consensus among researchers and clinicians as to whether the application of APC brackets can accelerate the bonding procedures in terms of time or reduce the failure rate during orthodontic treatments [34,35] (Table 8). In a clinical trial, Wong et al. [34] observed no difference in bonding time between APC and OPC brackets. In 80% of the cases, the bracket failure occurred in the first three months of treatment, with no significant difference in terms of bracket type. Similar results were achieved by Kula et al. [35], although APC brackets decreased the risk of failure when the bonding was performed by an unexperienced operator.

High SBS values may pose a risk for enamel during bracket removal at the end of the treatment. Indeed, a violation of tissue integrity can be undetectable without combined microscopic and spectroscopic methods. However, the loss of the enamel structure is invisible to the clinician’s naked eye, as it occurs at a microscopic scale and can result in oral biofilm adherence, hypersensitivity, and an increased risk of caries or fractures [1,25,26,27,28].

The majority of the quoted studies did not exhibit any significant differences in ARI scores between Metal-APC and Metal-OPC brackets when applied with the DB technique [26,28,29]. This paper addressed the issue of the combination of APC brackets with IDB, which had not been investigated in the literature previously [1,20]. In the present study, the mean ARI score in all tested groups did not exceed 2. Particular emphasis should be put on the fact that a smaller value of the ARI indicates stronger adhesion between the bracket and adhesive than between the enamel and adhesive. An ARI score of 2 is a compromise, indicating a strong adhesive connection to the enamel (more resistant to applied orthodontic forces during treatment) and reduced time required for enamel polishing. With an ARI score of 3, all the material left on the enamel surface implies longer and more time-consuming polishing procedures that can jeopardise the integrity of the enamel surface. With the increase in SBS, more adhesive remnants are left on the bracket surface; such a correlation between the SBS and ARI was statistically significant.

It is difficult to compare the ARI in various research studies because of the lack of standardisation; some authors used a modified ARI index (a five-grade scale) [36] instead of the basic Artun’s ARI score [24]. Hence, a correlation between the 3D profilometry (quantitative evaluation) results and the ARI score (qualitative) was proven [37]. Consequently, the ARI score can be applied as a general assessment of the remaining adhesive, while profilometry provides a more precise evaluation. To facilitate our analysis and for the purposes of this paper, the results found in the literature (presented according to the five-grade mARI scale) were converted into the unified zero–three-grade scale and are presented in Table 6 [8,25,26,28,29,35,36,38,39,40].

In the present study, specimens with BARI scores of 5 were investigated using SEM-EDS. They were chosen because this kind of adhesive failure is particularly dangerous for the enamel structure [6,7,11,23,35,36]. Although during macroscopic observation, no adhesive on the bracket base was detected, the SEM images showed not only the microstructure of the bracket base but also the presence of adhesive remnants. The adhesive resin covered the metal and APC base with a thin, inhomogeneous layer, blurring the image of the typical stainless steel. In the ceramic bracket, the smooth areas of resin were visible among disorganized crystal islets. Moreover, the EDS analysis revealed the presence of the following elements: Si, O, and C, as one would expect knowing the chemical composition of the adhesive. Namely, Transbond XT adhesive consists of ca. 23% organic matrix (Bis-GMA—C_29_H_36_O_8_) and 77% non-organic filler (quartz—SiO_2_) [33]. Presenting the post-debonding SEM images of APC brackets, Namura et al. [10] revealed the presence of inorganic filler particles diffused sparsely throughout the organic matrix. A similar microstructure was visible in the present study. SEM observations performed by Bishara et al. [38] confirmed the increased roughness of the ceramic bracket base in comparison to the metallic mesh base. This corresponds with the present results and can be one of the factors determining the increased SBS.

The EDS spectra of the bracket after debonding was particularly important since a combination of chemical components from the material and bracket was expected. In metal-APC and ceramic-OPC brackets, the presence of calcium was detected; the source of Ca was the enamel. The rationale of such results is that the tissue integrity was compromised during immediate debonding. However, in the metal-APC brackets, calcium ions were detected only in a trace values, while in the ceramic-OPC brackets, the presence of Ca was substantial. The analysis of the metal bracket base (with the lowest SBS value in the study groups) revealed no presence of Ca. Thus, a lower SBS (but still sufficient for orthodontic treatment) may be recommended in cases of congenital or acquired enamel defects [5,7,8,15,28,37,39,40,41,42,43].

Additionally, the enamel-safe removal of adhesive residuals is of crucial importance, as post-debonding polishing with a tungsten-carbide bur can cause micro-damage in the enamel. Similar findings were reported by Moecke et al. [39], who highlighted the consequences of microscopic enamel damage, such as an increased susceptibility to dental plaque accumulation and serious alterations in dental aesthetics.

The SEM images of the metal brackets that were not covered with adhesive revealed a rough surface as per the manufacturing process (metal injection moulding, sintering, and secondary thermal treatment) [42]. These brackets are made of austenitic stainless steel (300 series) and contain the following (depending on manufacturer): 69–72% Fe, 18–20% Cr, 8–12% Ni, 2%Mn, 1% Si 0.1% C, and less than 0.01% S, P, and Al. The EDS analysis confirmed this elemental composition. The ceramic polycrystalline brackets presented an inhomogeneous structure with different sizes and shapes of crystals. Their main component is Al_2_O_3_ and thus, Al and O were the only elements present in the EDS analysis. The disadvantage of EDS is the imprecision of quantitative analyses of carbon content. The source of carbon in the post-debonding bracket specimen might be the organic matrix of the adhesive material. Small amounts of carbon can also be detected in the metal bracket itself. Stainless-steel alloys include 0.03–0.15% wt., depending on the manufacturer [37,43]. However, carbon is most common surface contaminant in the SEM/EDS analysis, which affects the total carbon content detected in the sample. Thus, the percentage of C presented in Figure 12 cannot be interpreted as an exact amount. To obtain precise values, Zou et al. [44] recommend using field-emission SEM with a silicone drift detector, combined with quant optimization and a regression test prior to the specimen analysis.

In the present in vitro study, emphasis was placed on standardized conditions. The most important aspect was the selection of specimens. Although in some similar studies, extracted bovine teeth were used, this research was performed on human-borne dental tissues [19,43]. Differences in animal tissues’ microstructures could affect the SBS results. The choice of the upper first permanent premolar, without the unambiguous identification of the donors’ age, gender, and ethnicity, was also justified. Non-carious permanent premolars are frequently extracted due to orthodontic indications (i.e., lack of space in the dental arch). The extraction is usually performed in younger groups of patients, and age-associated changes in hard tissues are absent. The potential utility of the other types of teeth in SBS research is also questionable. For instance, the lower first incisor is usually extracted due to periodontal indications in older groups of patients. On the other hand, surgically extracted impacted third molars should be excluded despite of having untouched enamel—the hard tissues of such teeth are not exposed to fluoride and other elements in the oral cavity and might also be less mineralized.

The collected premolar specimens were thoroughly investigated to exclude any enamel imperfections, caries, or hypoplasia. Then, the teeth were stored (not longer than 6 weeks after extraction) in 10% formalin solution, because this preserving agent does not affect the SBS [19].

Intraoral material aging was imitated in vitro by thermocycling. The protocol presented in ISO TR 11450 (500 cycles at 5 and 55 °C) is regarded as inadequate to obtain a sufficient aging effect [37,43]. Researchers have suggested a minimum of 5000 cycles (5–55 °C) with a 60 s dwell time, and this regimen was implemented in the present study [37,43]. The standardized laboratory conditions provide comparable results, but they are not similar to the volatile intraoral environment. In vivo, the bracket–enamel bonding is subjected to the biofilm and the products of bacterial metabolism. The presence of saliva (whose composition is specific to the individual) can contaminate the surface of adherents and inhibit the adhesion. Additionally, the occlusal load exerted at the bracket is not rectilinear as the force is initiated with a universal testing machine. Thus, after thorough in vitro analyses, clinical trials must be performed.

The increased need for orthodontic treatment has triggered the introduction of quick, efficient, and enamel-safe bonding protocols. Bracket failure is considered as one of the most common aspects in prolonged treatments [5,40,41]; early bracket debonding occurs usually in 6–8% of cases [29]. Therefore, in the future, brackets should be tested after diverse etching and bonding combinations in order to find the protocol-dependent factors affecting the SBS. Brackets from various manufacturers as well as brackets with different mechanics (self-ligating or lingual attachments) should be included. The measurements should be performed not only on premolars, but also on other types of teeth using different time intervals after bonding. There is also a need to conduct more in vitro research to find the most enamel-safe post-debonding cleaning protocol.

There is the potential to implement further microscopic observations combined with spectrometry. 3D profilometry and atomic force microscopy provide the opportunity to obtain a quantitative analysis of debonded surfaces [45].

## 5. Conclusions

Despite the limitations of the present study, the following conclusions can be reached:

1. The type of bracket (metal/ceramic) has a significant effect on the SBS in the indirect bonding technique.
2. Ceramic-OPC brackets bonded indirectly to the enamel surface achieved the highest SBS; hence, the enamel damage was significantly higher than that of other brackets.
3. Metal-APC brackets bonded indirectly may represent the most appropriate choice due to the relatively high bond SBS and favourable debonding pattern.
4. Ceramic brackets can be an alternative for metal brackets in cases that require a high bond strength.
5. In the present study, the mean ARI score in all the tested groups did not exceed 2, which is a compromise between having a strong adhesive connection to the enamel (more resistant to applied orthodontic forces during treatment) and reduced time required for enamel polishing.
6. The debonding of orthodontic brackets with high SBS values can lead to enamel damage even when it is not noticeable clinically.
7. There is a need for the standardization of perimeters in in vitro orthodontic research.

## Figures and Tables

**Table 1:** Bonding protocol.

Application Area	Procedure
Bracket	Application of Transbond XT Light Cure Adhesive *
	Light curing (15 s)
Cast	Bracket positioning
	Preparation of transfer trays from Emiluma and Lumaloc and placing the cast into the water for 30 min
	Removal of transfer trays (with brackets inside)
Enamel	Etching (30 s), rinsing (30 s), and drying (30 s)
	Application of Sondhi Rapid Set System:
	Placing the tray
	Removal of the tray after material setting (2 min.)

* not applicable for APC brackets.

**Table 2:** Materials used in the study.

Material	Type of Material	Composition	Application Time	Area of Application
Scotchbond Universal Etchant (3M ESPE, St. Paul, MN, USA)	Acid	32% wg. H_3_PO_4_	30 s	Enamel (cleaned)
Transbond XT Primer (3M Unitek, Monrovia, CA, USA)	Polymerizable monomer	TEGDMA, Bis-GMA	NA	Enamel (etched, rinsed, dried)
Transbond XT (3M Unitek, Monrovia, CA, USA)	Light cure adhesive resin with quartz filler	Silane-treated quartz, Bis-GMA, silane; 77% quartz filler	NA	Bracket base
Sondhi Rapid Set Resin A (3M Unitek, Monrovia, CA, USA)	Chemical cure adhesive resin with silica filler	TEGDMA, Bis-GMA, silane treated silica, SbPh_3_, HQ	3–5 s per tooth followed by a gentle air burst for 1–2 s	Enamel (etched, rinsed)
Sondhi Rapid Set Resin B (3M Unitek, Monrovia, CA, USA)	Chemical cure adhesive resin	TEGDMA, Bis-GMA, silane treated silica, SbPh_3_, HQ, PTDE, dimethyl siloxane	NA	Bracket base
Emiluma (Ultradent Products Inc., South Jordan, UT, USA)	Silicone	Vinyl polisiloxane	NA	Cast
Lumaloc (Ultradent Products Inc., South Jordan, UT, USA)	Silicone	Quartz, silicone dioxide, tridecyl alcohol ethoxylated, dimethylsiloxane	Placed in water for 30 min after application	Cast (on the Emiluma layer)

H_3_PO_4_—phosphoric acid; TEGDMA—Triethylene Glycol Dimethacrylate; Bis-GMA—bisphenol A-glycidyl dimethacrylate; SbPh_3_ triphenylantimony; HQ—hydroquinone; PTDE—2,20-(p-tolylimino) diethanol; NA—not applicable.

**Table 3:** Brackets used in the study.

Study Group	Manufacturer	Bracket Type	Internal Structure	Prescription	Base Type	Base Area
Metal-OPC	Victory Series™ (3M, Unitek, Monrovia, CA, USA)	Metal	Austenitic stainless steel	.022″ MBT for upper premolar	Foil mesh	8.97 mm^2^
Metal-APC	APC Plus™ Victory Series™ (3M, Unitek, Monrovia, CA, USA)	Metal	Austenitic stainless steel	.022″ MBT for upper premolar	Foil mesh covered with light cure adhesive	8.97 mm^2^
Ceramic-OPC	Clarity™ Advanced (3M, Unitek, Monrovia, CA, USA)	Ceramic	Polycrystalline	.022″ MBT for upper premolar	Microcrystalline mechanical	11.69 mm^2^

**Table 4:** Comparison of BARI and ARI index.

Amount of Adhesive on Bracket Base [25]	BARI Score [25]	ARI Score [24]	Adhesive Left on the Tooth [24]
0%	1	3	100% (Distinct impression of the bracket mesh)
<25%	2	2	>50%
>25% and <50%	3	2	
>75%	4	1	<50%
100%	5	0	0%

**Table 5:** Chemical composition of bracket base before bonding (not covered with adhesive) Not applicable for APC brackets.

Bracket Type	Element	Weight%	Atomic%
Metal OPC	C	8.94	30.99
	Al	0.13	0.20
	Si	0.35	0.52
	Cr	15.61	12.50
	Mn	1.16	0.88
	Fe	70.07	52.26
	Ni	3.74	2.65
Ceramic-OPC	O	44.60	57.58
	Al.	55.40	42.42

**Table 6:** Chemical composition of bracket bases with BARI = 5.

Bracket Type	Element	Weight%	Atomic%
Metal-OPC	C	58.88	66.94
	O	34.96	29.84
	F	0.69	0.0
	Na	0.50	0.30
	Al	0.47	0.24
	Si	4.50	2.19
	P	0.00	0.00
Metal-APC	C	28.00	41.23
	O	27.83	30.76
	F	6.80	6.33
	Al.	7.09	4.64
	Si	21.97	13.83
	P	3.20	1.83
	Zn	5.12	1.38
Ceramic-OPC	C	39.40	47.59
	O	54.27	49.21
	Al	1.21	0.65
	Si	4.06	2.09
	P	0.67	0.32
	Ca	0.38	0.14

**Table 7:** Comparison of SBS between APC and OPC brackets in different studies (etch-and-rinse protocol, direct bonding).

Author	Number of Specimens	Type of Brackets	Conclusions
Guzman et al. (2013) [27]	90	Metal-OPC vs. Metal-APC	NSD
González-Serrano et al. (2019) [8]	120	Ceramic-OPC vs. Ceramic-APC	NSD
Marc et al. 2018 [29]	45	Ceramic-OPC vs. Ceramic-APC	NSD
Ansari et al. (2016) [30]	50	Ceramic-OPC vs. Ceramic-APC	SBS in APC significantly lower than in OPC
Lee et al. (2015) [31]	36	Ceramic-OPC vs. Ceramic-APC	SBS in APC significantly higher than in OPC
Abdelaziz (2020) [28]	96	Metal-OPC vs. Metal-APC	NSD between APC and OPC, SBS in ER significantly higher than in SE

NSD—no significant difference after debonding; ER—etch and rinse; SE—self-etch.

**Table 8:** Comparison of ARI scores between APC and OPC brackets in different studies.

Author (Year)	Type of Brackets	Mean mARI Converted to Artun’s Index	Interpretation	Author’s Conclusion
Lee et al. (2015) [31]	Ceramic APC Plus	2.66	>50% of adhesive left on the tooth in 100% APC specimens	NSD in ARI of APC Plus and OPC
	Ceramic OPC	2.58		
Ansari et al. (2016) [30]	Ceramic APC Flash Free	2.3	>50% of adhesive left on the tooth in 80% APC specimens	Both brackets achieved safe bracket failure pattern
	Ceramic OPC	2.8		
González-Serrano et al. (2019) [8]	Ceramic APC Flash Free	0.85 * without thermocycling	<50% of adhesive left on the tooth in 100% APC specimens	APC left significantly lower amount of adhesive on the tooth surface after debonding
	Ceramic OPC	1.75 * without thermocycling		
Abdelaziz (2020) [28]	Metal APC Plus, E&R	0.58	<50% adhesive left on the tooth in 80% APC specimens	NSD in ARI of APC and OPC
	Metal OPC, E&R	0.79		
Guzman et al. (2013) [27]	Metal ACP	1.47	<50% adhesive left on the tooth in 50% APC specimens	APC left significantly lower amount of adhesive than OPC
	Metal OPC	1.97		

*—specimens were not thermocycled.

## Data Availability

Data are contained within the article.

## Appendix A

**Table A1:** Results of Tukey’s test for study groups.

Group	Difference	SE	*p*-Value
Metal-OPC vs. Metal-APC	3.542	0.5352	0.0002
Metal-OPC vs. Ceramic-OPC	4.819	0.5352	0.0000
Metal-APC vs. Ceramic-OPC	1.277	0.5352	0.2249

n = 10; *p*-values were rounded up to the fourth decimal place.
